# Oral zinc for treating diarrhoea in children

**DOI:** 10.1590/S1516-31802011000200013

**Published:** 2011-03-03

## Abstract

**BACKGROUND:**

Diarrhoea causes around two million child deaths annually. Zinc supplementation could help reduce the duration and severity of diarrhoea, and is recommended by the World Health Organization and UNICEF.

**OBJECTIVE:**

To evaluate oral zinc supplementation for treating children with acute or persistent diarrhoea.

**CRITERIA FOR CONSIDERING STUDIES FOR THIS REVIEW:**

In November 2007, we searched the Cochrane Infectious Diseases Group Specialized Register, CENTRAL (The Cochrane Library 2007, Issue 4), MEDLINE, EMBASE, LILACS, CINAHL, mRCT, and reference lists. We also contacted researchers.

**SELECTION CRITERIA:**

Randomized controlled trials comparing oral zinc supplementation (≥ 5 mg/day for any duration) with placebo in children aged one month to five years with acute or persistent diarrhoea, including dysentery.

**DATA COLLECTION AND ANALYSIS:**

Both authors assessed trial eligibility and methodological quality, extracted and analysed data, and drafted the review. Diarrhoea duration and severity were the primary outcomes. We summarized dichotomous outcomes using risk ratios (RR) and continuous outcomes using mean differences (MD) with 95% confidence intervals (CI). Where appropriate, we combined data in meta-analyses (using the fixed- or random-effects model) and assessed heterogeneity.

**MAIN RESULTS:**

Eighteen trials enrolling 6165 participants met our inclusion criteria. In acute diarrhoea, zinc resulted in a shorter diarrhoea duration (MD −12.27 h, 95% CI −23.02 to −1.52 h; 2741 children, 9 trials), and less diarrhoea at day three (RR 0.69, 95% CI 0.59 to 0.81; 1073 children, 2 trials), day five (RR 0.55, 95% CI 0.32 to 0.95; 346 children, 2 trials), and day seven (RR 0.71, 95% CI 0.52 to 0.98; 4087 children, 7 trials). The four trials (1458 children) that reported on diarrhoea severity used different units and time points, and the effect of zinc was less clear. Subgroup analyses by age (trials with only children aged less than six months) showed no benefit with zinc. Subgroup analyses by nutritional status, geographical region, background zinc deficiency, zinc type, and study setting did not affect the results' significance. Zinc also reduced the duration of persistent diarrhoea (MD −15.84 h, 95% CI −25.43 to −6.24 h; 529 children, 5 trials). Few trials reported on severity, and results were inconsistent. No trial reported serious adverse events, but vomiting was more common in zinc-treated children with acute diarrhoea (RR 1.71, 95% 1.27 to 2.30; 4727 children, 8 trials).

**AUTHORS' CONCLUSIONS:**

In areas where diarrhoea is an important cause of child mortality, research evidence shows zinc is clearly of benefit in children aged six months or more.

**PLAIN LANGUAGE SUMMARY:**

In developing countries, millions of children suffer from severe diarrhoea every year. This is due to infection and malnutrition, and many die from dehydration due to the diarrhoea. Giving fluids by mouth (using an oral rehydration solution) has been shown to save children's lives, but it seems to have no effect on the length of time the children suffer with diarrhoea. Children in developing countries are often zinc deficient. Zinc supplementation is a possible treatment for diarrhoea though it can have adverse effects if given in high doses. The review of trials identified 18 trials involving 6165 children of all ages. Zinc reduced the time that children over the age of six months suffered from symptoms of acute or persistent diarrhoea. However, there were insufficient data to see any impact on the number of children who died. More children vomited when given zinc, but it was considered that the benefits outweighed these adverse effects. Zinc seemed to have no impact on children aged less than six months. In areas where diarrhoea is an important cause of child mortality, research evidence shows zinc is clearly of benefit in children aged six months or more with diarrhoeal diseases.

**FURTHER INFORMATION** Brazilian Cochrane Center Rua Pedro de Toledo, 598 Vila Clementino — São Paulo (SP) — Brasil CEP 04039–001 Tel. (+55 11) 5575–2970 Email: cochrane.dmed@unifesp.br http:www.centrocochranedobrasil.org.br

## COMMENTS

During the 20th century, oral rehydration therapy for children suffering from diarrhea was developed and applied in an effective manner. This has been considered to be the therapeutic method that singly saved the largest number of lives during that century. Thus, the two pillars of treatment for acute diarrhea and persistent diarrhea (i.e. the form that begins with an episode of acute diarrhea, of presumably infectious origin, and extends for longer than 14 days, in children less than five years of age, and brings about negative repercussions on nutritional status) became solidified: 1. oral rehydration therapy; and 2. prevention and combating of malnutrition through adequate feeding.

Starting from this point, space was created such that the main therapeutics for acute diarrhea were broadened towards additional objectives, especially reduction of the duration of diarrhea and reduction of abnormal fecal losses. To this end, clinical research focused on the therapeutic role of zinc, probiotics and intestinal secretion reducers such as racecadotril, bismuth subsalicylate and vitamin A, among others. These therapeutic measures, used in conjunction with nutritional care and oral rehydration, were recently analyzed in detail in the Guidelines of the European Society for Pediatric Gastroenterology, Hepatology and Nutrition ^1^ and in the Iberian-Latin American Clinical Practice Guide.^2^

The systematic review and meta-analysis by Lazzerini and Ronfani ^3^ demonstrated that zinc administration is associated with mean reductions in the duration of acute diarrhea and persistent diarrhea of 12.3 hours and 15.8 hours, respectively, for children between the ages of six months and five years. Considering that the clinical trials were carried out in underdeveloped countries, the authors concluded that in areas in which diarrhea is an important cause of mortality among infants and young children, the scientific evidence indicates that zinc therapy is efficient. Furthermore, the World Health Organization (WHO) considers that zinc reduces the future risk of a new diarrhea outbreak during the subsequent two to three months. The addition of such measures within the Brazilian public health scenario requires a cost-effectiveness analysis that takes into consideration the need for preparation and distribution of zinc-containing products, in the same way in which oral rehydration salts were investigated in the past.
